# Early survival for patients newly diagnosed with cancer during COVID‐19 in Ontario, Canada: A population‐based cohort study

**DOI:** 10.1002/cam4.5861

**Published:** 2023-03-31

**Authors:** Rui Fu, Rinku Sutradhar, Qing Li, Pabiththa Kamalraj, Anna Dare, Timothy P. Hanna, Kelvin K. W. Chan, Jonathan C. Irish, Natalie Coburn, Julie Hallet, Simron Singh, Ambica Parmar, Craig C. Earle, Lauren Lapointe‐Shaw, Monika K. Krzyzanowska, Alexander V. Louie, Alyson Mahar, David R. Urbach, Daniel I. McIsaac, Danny Enepekides, David Gomez, Nicole J. Look Hong, Jill Tinmouth, Antoine Eskander

**Affiliations:** ^1^ ICES Toronto Ontario Canada; ^2^ Institute of Health Policy, Management, and Evaluation, Dalla Lana School of Public Health University of Toronto Toronto Ontario Canada; ^3^ Department of Otolaryngology–Head and Neck Surgery University of Toronto Toronto Ontario Canada; ^4^ Department of Surgery University of Toronto Toronto Ontario Canada; ^5^ Division of Cancer Care and Epidemiology, Cancer Research Institute Queen's University Kingston Ontario Canada; ^6^ Ontario Institute for Cancer Research (OICR) Toronto Ontario Canada; ^7^ Odette Cancer Centre–Sunnybrook Health Sciences Centre Toronto Ontario Canada; ^8^ Ontario Health–Cancer Care Ontario Toronto Ontario Canada; ^9^ Department of Otolaryngology–Head & Neck Surgery/Surgical Oncology University of Toronto, Princess Margaret Cancer Centre Toronto Ontario Canada; ^10^ Department of Medicine University of Toronto Toronto Ontario Canada; ^11^ Department of Radiation Oncology University of Toronto Toronto Ontario Canada; ^12^ School of Nursing Queen's University Kingston Ontario Canada; ^13^ Department of Surgery Women's College Hospital Toronto Ontario Canada; ^14^ Department of Anesthesiology and Pain Medicine The Ottawa Hospital Ottawa Ontario Canada; ^15^ Division of General Surgery, St. Michael's Hospital Unity Health Toronto Toronto Ontario Canada

**Keywords:** cancer, cancer detection, cancer survivorship, COVID‐19, oncology

## Abstract

**Background:**

Little is known about the association between the COVID‐19 pandemic and early survival among newly diagnosed cancer patients.

**Methods:**

This retrospective population‐based cohort study used linked administrative datasets from Ontario, Canada. Adults (≥18 years) who received a cancer diagnosis between March 15 and December 31, 2020, were included in a pandemic cohort, while those diagnosed during the same dates in 2018/2019 were included in a pre‐pandemic cohort. All patients were followed for one full year after the date of diagnosis. Cox proportional hazards regression models were used to assess survival in relation to the pandemic, patient characteristics at diagnosis, and the modality of first cancer treatment as a time‐varying covariate. Interaction terms were explored to measure the pandemic association with survival for each cancer type.

**Results:**

Among 179,746 patients, 53,387 (29.7%) were in the pandemic cohort and 37,741 (21.0%) died over the first post‐diagnosis year. No association between the pandemic and survival was found when adjusting for patient characteristics at diagnosis (HR 0.99 [95% CI 0.96–1.01]), while marginally better survival was found for the pandemic cohort when the modality of treatment was additionally considered (HR 0.97 [95% CI 0.95–0.99]). When examining each cancer type, only a new melanoma diagnosis was associated with a worse survival in the pandemic cohort (HR 1.25 [95% CI 1.05–1.49]).

**Conclusions:**

Among patients able to receive a cancer diagnosis during the pandemic, one‐year overall survival was not different than those diagnosed in the previous 2 years. This study highlights the complex nature of the COVID‐19 pandemic impact on cancer care.

## Graphical abstract

This population‐based cohort study in Ontario, Canada found no association between the pandemic and the 1‐year overall survival of adults newly diagnosed with cancer. These results highlight the complex nature of the pandemic impact on cancer care.

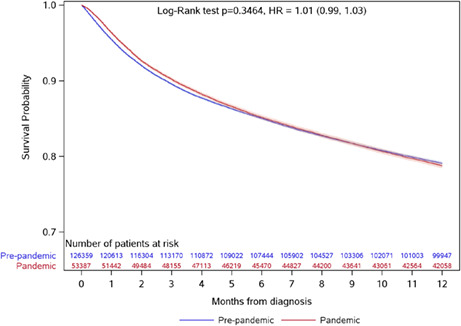

## 1 INTRODUCTION

The COVID‐19 pandemic has transformed almost all aspects of cancer care, most notably with reduced access to cancer screening, diagnostic imaging, and treatment initiation.^1^, ^2^, ^3^, ^4^ Concerns about the direct adverse impact of the pandemic on short‐term survival have been raised, with a number of different hypotheses. Cancer patients are already vulnerable to COVID‐19 infection and death, and those who recently underwent anti‐cancer treatment (particularly chemotherapy) may have an even higher risk of dying from COVID‐19.^5^ For others awaiting treatment, even a 4‐week delay could increase mortality in 7 major cancer types,^6^ while COVID‐19 lockdown have delayed treatment for many lower‐priority patients by more than 4 weeks.^7^ During the emergency cessation of surgical services, surgical resection was replaced by systemic therapy/radiotherapy, with limited data showing whether patients ultimately received surgery or died prematurely.^8^, ^9^ When cancer screening was suspended and in‐person physician examinations were minimized, patients presenting to hospital with cancer tended to be those suffering from advanced‐stage disease, leading to higher rates of death during the pandemic.^10^

Modeling studies have projected the pandemic would cause excess cancer mortality in the next 5–20 years,^11^, ^12^, ^13^, ^14^ but the question remains whether the pandemic has already been associated with a decrease in real‐world survival for newly diagnosed patients. Several studies have assessed this question and provided conflicting early results.^10^, ^15^, ^16^, ^17^, ^18^, ^19^, ^20^, ^21^, ^22^, ^23^, ^24^, ^25^, ^26^, ^27^, ^28^ Shared limitations of these studies included small sample size, focus on a single cancer disease site, and incomplete patient follow‐up. Hence, our objective was to comprehensively examine whether the pandemic was associated with the overall survival of adults diagnosed with major cancer types during the first year after diagnosis. The results of this analysis will provide powerful data to support modeling studies and to aid the formulation of post‐pandemic recovery strategies.

## 2 METHODS

### 2.1 Study design

This was a retrospective population‐based cohort study in Ontario, Canada where 14.7 million residents access healthcare services under a single‐payer system, the Ontario Health Insurance Plan (OHIP). Administrative datasets were linked using unique encoded identifiers and analyzed at ICES. Ethical approval was not sought, as the use of data was approved by ICES' Privacy and Legal Office. This study followed the Reporting of studies Conducted using Observational Routinely collected health data (RECORD) and the Strengthening the Reporting of Observational Studies in Epidemiology (STROBE) guidelines. The analysis was conducted between June–July 2022.

### 2.2 Data sources

The Ontario Cancer Registry (OCR) is a population‐based registry capturing 96% of cancer diagnoses in the province.^29^ The Registered Persons Database (RPDB) maintains demographic information on individuals covered under OHIP. The Immigration, Refugees and Citizenship Canada (IRCC) Permanent Resident Database (with data from January 1985 to May 2017) includes records of individuals who immigrated to Ontario during this period. Statistics Canada's Postal Code Conversion File (PCCF) contains the status of rural residence.^30^ The Ontario Marginalization Index (ON‐MARG) database quantifies material deprivation.^31^ Data holdings at the Canadian Institute of Health Information (CIHI) including the Same‐Day Surgery and the Discharge Abstract Databases identify cancer‐directed surgeries performed at a hospital. The OHIP claims database stores physician billing records (Appendix I).

### 2.3 Study cohort

We created a pandemic cohort, consisting of adults (≥18 years) with an invasive cancer diagnosis from March 15 to December 31, 2020, and a pre‐pandemic cohort, consisting of those diagnosed during the corresponding periods in 2019 and 2018. March 15, 2020, was when hospitals in Ontario were advised by the Chief Medical Officer of Health to discontinue nonemergent and elective procedures.^32^ December 31, 2020, reflects the last reliable data update from the OCR at the time of this analysis. Invasive cancer diagnoses were identified using International Classification of Diseases for Oncology, third edition codes with malignant morphology in the OCR (Appendix II). If more than one invasive cancer diagnosis occurred for the same patient during the three yearly time intervals, only the earliest diagnosis was selected (Figure S1). We excluded patients who had more than one invasive cancer type diagnosed on the same day. We also excluded those with a date of cancer diagnosis that coincided with the date of death (*n* = 1624, 0.9%) to mitigate the impact of an expected drop in the number of patients whose cancer diagnosis was documented by death certificate during the pandemic; a sensitivity analysis was later performed to further examine this issue. The date of death was obtained from the RPDB, a validated source of vital statistics that was updated until April 2022 at the time of this analysis. For patients in the final cohort, they were followed from the date of cancer diagnosis for 1 year (365 days) or until the date of death from any cause, whichever happened first. Hence, a complete 1‐year follow‐up window was available for each patient.

### 2.4 Outcome

The outcome of this study was overall survival within 1 year of cancer diagnosis, a time‐to‐event variable capturing the number of days from cancer diagnosis (index date) to death. Patients who were alive at the end of the first year after diagnosis were censored at that point.

### 2.5 Covariates

The following patient characteristics were measured at the time of cancer diagnosis. Age and sex were obtained from the RPDB. Rural residence was defined as living in a small town or rural area with a population of less than 10,000 in the PCCF.^30^ Status of having immigrated to Ontario from January 1985 to May 2017 was determined from the IRCC Permanent Resident Database. Material deprivation was reported in quintiles.^31^ Comorbidity was calculated by the Elixhauser Comorbidity Index using hospitalization records in 5 years preceding the cancer diagnosis.^33^ Five comorbidity groups were created for patients who scored 0, 1, 2, ≥3 on the index and for those who were not hospitalized. Cancer type was abstracted from OCR diagnostic records.

For each patient, we followed an established algorithm to determine the modality of first cancer treatment: surgery, systemic therapy, radiotherapy, or being untreated over the first post‐diagnosis year.^4^, ^9^, ^34^, ^35^ Cancer surgical resection performed at a hospital was identified first using surgical procedure codes from CIHI and then matched to OCR diagnosis records with respect to surgical site and cancer type; surgical biopsies were excluded. Both systemic therapy and radiotherapy visits were derived from the OHIP claims database. We restricted systemic therapy to be physician‐supervised intravenous infusions since oral agents (such as hormonal therapy) were not robustly captured in the physician billing database.^8^

### 2.6 Statistical analysis

We compared the characteristics of the pre‐pandemic and the pandemic cohort using 0.10 as a standardized difference threshold.^36^ We conducted a Kaplan–Meier analysis to compare the survival of the two cohorts using a log‐rank test. To measure the unadjusted association between the pandemic and survival, we used a univariable Cox proportional hazards regression model including only the pandemic indicator variable. A multivariable Cox model (Model 1) was then used to account for all fixed covariates (except treatment) chosen a priori based on the literature.^3^, ^34^, ^37^ A second multivariable model (Model 2) was then fit by adding treatment modality as a categorical 4‐level time‐varying covariate to Model 1. This means that a patient would only move into their specific treatment category once they experienced this treatment. To estimate the pandemic association with survival for each cancer type, we used a third multivariable model (Model 3) by adding 2‐way interactions between cancer type and the pandemic indicator to Model 2. In a sensitivity analysis, we repeated all procedures on the full cohort including those who died on the date of cancer diagnosis. All analyses were two‐sided, and statistical significance was set at *p*‐value<0.05. Analyses were performed on SAS Enterprise Guide 7.15 (SAS Institute).

## 3 RESULTS

Of the 179,746 patients included, 126,359 (70.3%) patients were in the pre‐pandemic cohort and 53,387 (29.7%) were in the pandemic cohort. At the time of cancer diagnosis, there were no significant differences in sociodemographic and clinical characteristics between the two cohorts (Table 1). Over the first year after the diagnosis (Table 2), the modality of first cancer treatment also did not differ between the two cohorts. Among those who were treated within the first year (*n* = 135,828, 75.6%), the mean time‐to‐treatment was 6 days shorter in the pandemic cohort, dropping from 51.5 days (SD 57.5) to 45.6 days (SD 52.7, standardized difference 0.11).

**TABLE 1:** Characteristics of patients at time of cancer diagnosis (*n* = 179,746).

Variables	Pre‐pandemic cohort (*n* = 126,359)	Pandemic cohort (*n* = 53,387)	Standardized difference
Age at diagnosis, y, Mean ± SD	66.68 ± 14.08	66.43 ± 14.16	0.02
Female	64,052 (50.7%)	27,208 (51.0%)	0.01
Rural^a^	16,244 (12.9%)	7058 (13.2%)	0.01
Immigrant	16,020 (12.7%)	7013 (13.1%)	0.01
Region			
Central	36,577 (28.9%)	15,390 (28.8%)	0
East	32,680 (25.9%)	13,870 (26.0%)	0
North	8837 (7.0%)	3887 (7.3%)	0.01
Toronto	10,004 (7.9%)	4055 (7.6%)	0.01
West	38,261 (30.3%)	16,185 (30.3%)	0
Material deprivation quintile^a^ ^,^ ^b^			
First, least deprived	27,590 (21.8%)	11,742 (22.0%)	0
Second	25,743 (20.4%)	11,029 (20.7%)	0.01
Third	24,536 (19.4%)	10,322 (19.3%)	0
Fourth	23,930 (18.9%)	10,097 (18.9%)	0
Fifth, most deprived	23,492 (18.6%)	9722 (18.2%)	0.01
Comorbidity grouping^c^			
0	12,077 (9.6%)	5313 (10.0%)	0.01
1	10,234 (8.1%)	4430 (8.3%)	0.01
2	7607 (6.0%)	3094 (5.8%)	0.01
≥3	10,916 (8.6%)	4313 (8.1%)	0.02
No hospitalization	85,525 (67.7%)	36,237 (67.9%)	0
Cancer type			
Breast	18,193 (14.4%)	7654 (14.3%)	0
Central nervous system	1563 (1.2%)	759 (1.4%)	0.02
Cervical	972 (0.8%)	423 (0.8%)	0
Colorectal	13,829 (10.9%)	5868 (11.0%)	0
Endocrine	4613 (3.7%)	1780 (3.3%)	0.02
Esophagus	1326 (1.0%)	588 (1.1%)	0.01
Genitourinary	8955 (7.1%)	3862 (7.2%)	0.01
Gynecologic excluding cervical	6896 (5.5%)	3215 (6.0%)	0.02
Head and neck	3974 (3.1%)	1808 (3.4%)	0.01
Hepatobiliary	6313 (5.0%)	2823 (5.3%)	0.01
Lung	15,513 (12.3%)	6442 (12.1%)	0.01
Lymphoma	6441 (5.1%)	3017 (5.7%)	0.02
Melanoma	6531 (5.2%)	2353 (4.4%)	0.04
Ophthalmologic	53 (0.0%)	16 (0.0%)	0.01
PNS	21 (0.0%)	10 (0.0%)	0
Prostate	14,119 (11.2%)	5270 (9.9%)	0.04
Sarcoma	1978 (1.6%)	883 (1.7%)	0.01
Skin	442 (0.3%)	137 (0.3%)	0.02
Stomach	2182 (1.7%)	938 (1.8%)	0
Other	12,445 (9.8%)	5541 (10.4%)	0.02

*Note*: Data are *n* (%) or mean ± standard deviation (for age). The pandemic cohort included adult patients diagnosed with cancer during March 15–December 31, 2020, to coincide with the first 9.5 months since hospitals had been advised to discontinue nonemergent and elective procedures. Corresponding dates in 2018 and 2019 were used to construct a pre‐pandemic cohort. A standardized difference of 0.1 or greater indicated a significant imbalance in the distributions.

Abbreviation: PNS, Paraneoplastic neurological syndromes.

^a^ Missing data of these variables were <1.0% of the study cohort and the distributions of missingness did not differ by cohort (both standardized differences = 0).

^b^ Material deprivation is a composite measure of socioeconomic status that includes the proportion of the population that is without a high school degree, single‐parent families, unemployed, low‐income, receiving government transfer payments, and living in a dwelling in need of a major repair.

^c^ The Elixhauser Comorbidity Index was computed based on health administrative data 5 years leading to the date of cancer diagnosis.

**TABLE 2:** First cancer treatment received within the first year after diagnosis.

Variables	Pre‐pandemic cohort (*n* = 126,359)	Pandemic cohort (*n* = 53,387)	Standardized difference
First cancer treatment			
Untreated	32,267 (25.5%)	11,651 (21.8%)	0.09
Systemic therapy	21,826 (17.3%)	10,938 (20.5%)	0.08
Radiotherapy	19,139 (15.1%)	8828 (16.5%)	0.04
Surgery	53,127 (42.0%)	21,970 (41.2%)	0.02
Time to first treatment, day			
Mean ± SD	51.5 ± 57.5	45.6 ± 52.7	0.11
Median (IQR)	37.0 (15.0–66.0)	33.0 (13.0–58.0)	0.11

*Note*: Time to first treatment was only calculated for the sub‐cohort of patients that was treated by any means within the first year after cancer diagnosis. The pandemic cohort comprised patients diagnosed with cancer from March 15 to December 31, 2020, and the pre‐pandemic cohort included patients diagnosed during the corresponding dates in 2018 and 2019.

Abbreviations: IQR, interquartile range; SD, standard deviation.

**FIGURE 1:**
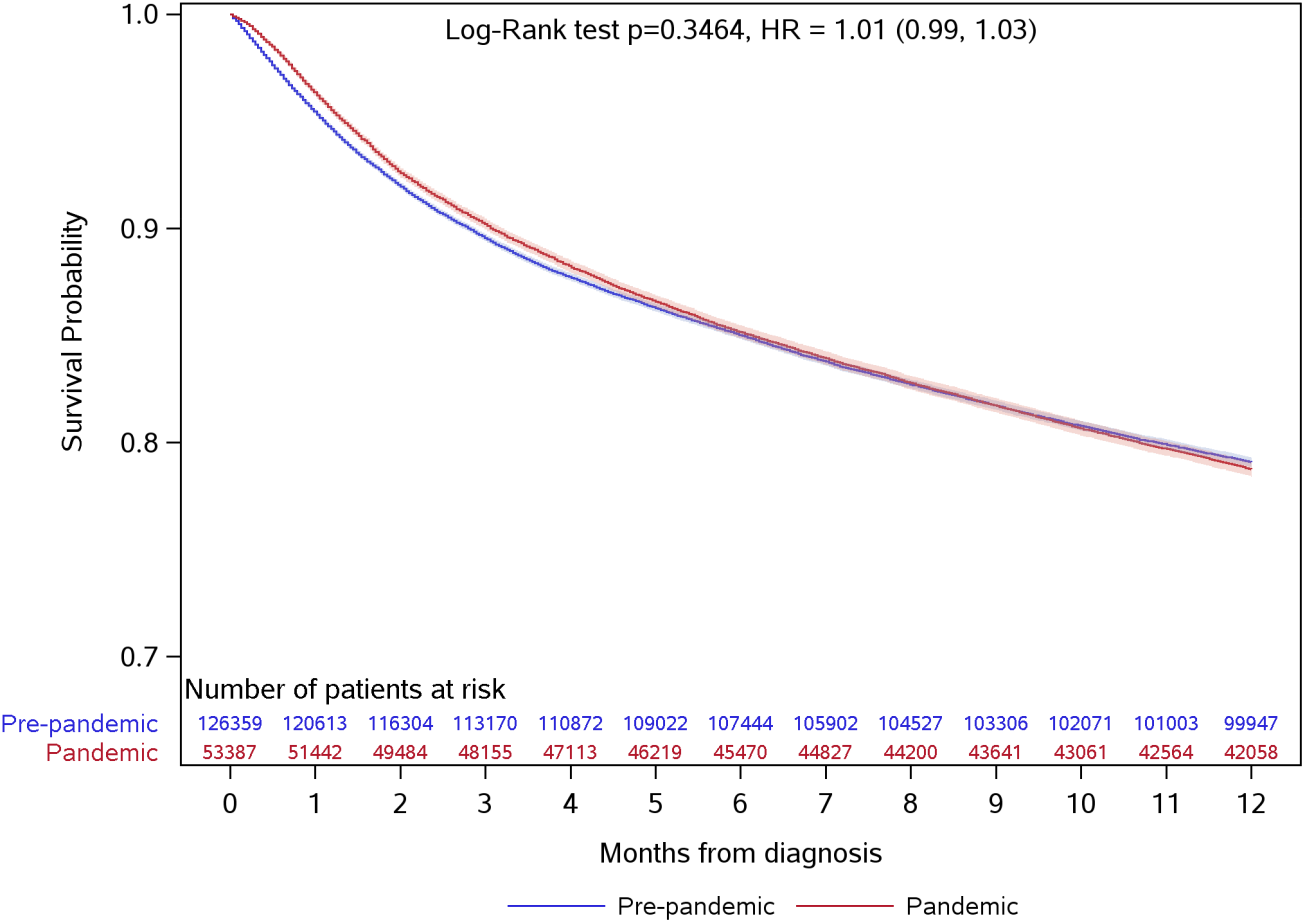
Survival in new cancer patients within the first year after diagnosis (*n* = 179,746). Adult patients diagnosed with cancer during March 15–December 31, 2020, the first 9.5 months of pandemic control in Ontario, Canada, were included in the pandemic cohort, whereas those diagnosed during the corresponding dates in 2018 and 2019 were included in the pre‐pandemic cohort.

A full 1‐year of data was available on all patients for capturing mortality. At the end of the first year after diagnosis, 37,741 (21.0%) patients in the entire cohort died. The Kaplan–Meier plot (Figure 1) demonstrated the two cohorts of patients had a similar survival over the first year after diagnosis (log‐rank *p*‐value = 0.35). Specifically, the estimated 1‐year survival probability was 78.7% (95% CI: 78.4%–79.1%) for the pandemic cohort, compared to 78.1% (95% CI: 77.9%–78.3%) for the pre‐pandemic cohort.

Cox regression models were used to quantify the association of survival with the pandemic (Table 3). In the univariable analysis, we found no significant associations (HR 1.01, 95% CI 0.99–1.03). This persisted after adjusting for patient covariates at diagnosis (Model 1; aHR 0.99, 95% CI 0.96–1.01). By additionally considering the modality of first cancer treatment as a time‐varying covariate (Model 2), the pandemic cohort was associated with a marginally higher survival (aHR 0.97, 95% CI 0.95–0.99) than the pre‐pandemic cohort.

**TABLE 3:** Results of the Cox regression analysis showing the association between 1‐year overall survival and the pandemic (*n* = 179,746).

Variables	HR	95% CI	*p*‐value
Univariable model			
Pre‐pandemic	Reference		
Pandemic	1.01	0.99–1.03	0.35
Multivariable model (Model 1)			
Pre‐pandemic	Reference		
Pandemic	0.99	0.96–1.01	0.18
Multivariable model (Model 2)			
Pre‐pandemic	Reference		
Pandemic	0.97	0.95–0.99	<0.01
Status of first cancer treatment			
Untreated	Reference		
Systemic therapy	1.35	1.31–1.40	<0.01
Radiotherapy	1.66	1.61–1.72	<0.01
Surgery	0.42	0.41–0.44	<0.01
Age at cancer diagnosis, each 10‐year increase	1.49	1.47–1.50	<0.01
Male	Reference		
Female	0.93	0.91–0.95	<0.01
Urban residence	Reference		
Rural residence	1.07	1.04–1.10	<0.01
Non‐immigrants	Reference		
Immigrants	0.84	0.81–0.87	<0.01
Material deprivation			
Fifth quintile, most deprived	Reference		
Fourth	0.91	0.88–0.94	<0.01
Third	0.87	0.85–0.90	<0.01
Second	0.82	0.80–0.85	<0.01
First quintile, least deprived	0.77	0.75–0.80	<0.01
Comorbidity			
No hospitalization	Reference		
0	1.01	0.97–1.05	0.61
1	1.16	1.12–1.20	<0.01
2	1.26	1.22–1.31	<0.01
≥3	1.51	1.47–1.55	<0.01
Cancer type			
Breast	Reference		
Colorectal	3.81	3.55–4.09	<0.01
Endocrine	1.27	1.07–1.50	<0.01
Esophagus	7.96	7.28–8.71	<0.01
Genitourinary	3.95	3.66–4.27	<0.01
Gynecologic excluding cervical	3.22	2.96–3.50	<0.01
Head and neck	3.00	2.75–3.28	<0.01
Hepatobiliary	12.14	11.33–13.01	<0.01
Lung	7.21	6.75–7.71	<0.01
Lymphoma	2.99	2.77–3.24	<0.01
Melanoma	1.16	1.05–1.28	<0.01
Prostate	0.65	0.60–0.72	<0.01
Sarcoma	2.73	2.45–3.05	<0.01
Skin	1.64	1.33–2.02	<0.01
Stomach	7.70	7.09–8.36	<0.01
Other^a^	4.39	4.10–4.71	<0.01

*Note*: Model 1 adjusts for all covariates in Model 2 except for first cancer treatment modality as a time‐varying covariate.

Abbreviations: HR, hazard ratio; CI, confidence interval.

^a^ Other includes cancers of the central nervous system, cervix, eyes, paraneoplastic neurological syndromes, and those with ill‐defined or unknown primary sites.

Interaction between the pandemic and each cancer type was added to Model 2 (Model 3). In Appendix III, we report the pandemic association with survival to be significantly different across cancer types (Type‐III *p*‐value<0.01). Specifically, hepatobiliary (aHR 0.85, 95% CI 0.80–0.90) and lung (aHR 0.95, 95% CI 0.91–0.99) cancers were associated with a higher survival for the pandemic cohort, while melanoma was associated with a worse survival for the pandemic cohort (aHR 1.25, 95% CI 1.05–1.49).

Results from the sensitivity analysis are presented in Appendix IV. The full cohort (*n* = 181,370) now included those who died on the date of cancer diagnosis (*n* = 1624). Again, we observed no difference in patient characteristics at cancer diagnosis between the two cohorts (Table S1), although fewer patients remained untreated over the first year in the pandemic cohort than in the pre‐pandemic cohort (21.8% vs. 26.5%, standardized difference, 0.11; Table S2). The Kaplan–Meier plot (Figure S2) depicted a significant 1‐year survival difference favoring the pandemic cohort (log‐rank *p*‐value<0.01). The higher survival persisted in all three Cox models (HRs ranging from 0.92–0.96, all *p*‐value<0.01; Table S3). When inspecting each cancer type (Table S4), hepatobiliary and lung cancers were again associated with higher survival in the pandemic cohort, while colorectal (aHR 0.93, 95% CI 0.87–0.999) and other (aHR 0.93, 95% CI 0.88–0.98) rarer cancers (such as cancers of the central nervous system) were also associated with a higher survival in the pandemic cohort. Melanoma remained the only cancer type associated with a worse survival in the pandemic cohort (aHR 1.23, 95% CI 1.03–1.46).

## 4 DISCUSSION

In this population‐based cohort study, the 1‐year survival of adult patients diagnosed with cancer during the pandemic was not statistically different from those diagnosed in the past 2 years. Melanoma was the only cancer type showing a worse survival during the pandemic.

To our best knowledge, this is the largest study to date that examines the pandemic association with survival in new patients of all major cancer types (Appendix V). Compared to the two existing studies, both using single‐center designs, a similar lack of change in survival during the pandemic was observed in Portugal.^18^ Conversely, a Turkish tertiary medical oncology center reported a decrease in 90‐day survival.^10^ This discrepancy may be attributed to the large 28% reduction in new patient referrals at this center, which implies a pronounced pandemic‐induced patient selection whereby only those requiring urgent medical attention were referred and died shortly afterward. Furthermore, this center was predominately involved in advanced‐stage disease management since 80% of its patients required palliative radiotherapy at first referral. With this specific patient case‐mix, the elevated death rate might also be explained by their vulnerability to COVID‐19‐related mortality. Future study focusing on the provision of palliative oncologic care during the pandemic is required to assess this hypothesis.

Despite an overall unchanged survival, we conducted a focused investigation to identify if certain cancer types had indeed demonstrated a decrease in survival during the pandemic. For colorectal cancer, we ruled out such negative pandemic association, which concurs with all previous studies.^15^, ^20^, ^21^, ^22^, ^24^, ^26^ However, these studies were limited by an incomplete capture of mortality data and were subject to residual confounding as they did not account for time‐to‐treatment.^6^ We improved upon this by including time‐to‐treatment as a time‐varying covariate, and as such, strengthened the conclusion on a comparable survival for newly diagnosed colorectal cancer patients in early pandemic.

Among patients who presented with hepatobiliary or lung cancer during the pandemic, their 1‐year survival was marginally better than those who presented before the pandemic. These findings are in line with another Canadian single‐center study^25^ and studies based in the United Kingdom,^23^ Spain,^16^ and France.^17^ However, a US study on metastatic pancreatic cancer suggested a worse 180‐day survival for those diagnosed during the pandemic.^19^ We attribute this discrepancy to the advanced‐stage status of the US cohort and differences between the United States and Canadian healthcare systems. Our prior work suggested the weekly incidence of these cancers not only dropped as soon as the pandemic started, but it also continued to decrease up to September 2020.^37^ Such sustained reduction in care demand might imply that when compared to the pre‐pandemic, newly diagnosed patients have had more expeditious access to treatment^4^ and even more comprehensive care including virtual psychosocial care.^1^ This may explain the stable, if not slightly better, survival in those able to get a hepatobiliary or lung cancer diagnosis.

We found melanoma to be the only cancer type associated with a worse short‐term survival during the pandemic. This is potentially concerning as melanoma mortality rates have been decreasing consistently in the past decade, thanks to advances in targeted therapies and immunotherapy.^38^ Since melanoma can spread rapidly to other organs, the worse survival may be attributed to an upstage migration. Specifically, incidental detection of early‐stage melanoma has decreased during the pandemic,^39^ which, coupled with the large drop in skin biopsies and in‐person dermatologist visits, has shifted the stage distribution of melanoma to more advanced disease.^40^ More research is required to pinpoint which components in the structure or process of melanoma care were responsible for the decrease in survival.

Many factors may have contributed to the observed survival of patients diagnosed with cancer during the pandemic. Partially, this may reflect the short‐term success of the pandemic control policy, particularly the directed prioritization of cancer surgical care over non‐oncologic elective surgeries, the expanded use of neoadjuvant therapy, and the rapid adoption of telemedicine.^1^, ^3^, ^4^ The long‐term consequences of these mitigation strategies warrant future research. The stability of 1‐year survival may also be a result of shifts in the patient case‐mix, particularly related to stage. For instance, some advance‐stage patients who would have died of their cancer instead died of COVID‐19 prior to receiving a cancer diagnosis. The non‐inferior early survival might also be attributed to a decrease in acute postoperative complications (due to less cancer surgery being performed^34^, ^35^), more stringent hygiene standards in the public, and better health practice at home.^41^

It is worth noting, however, that these results only apply to patients who were able to get a cancer diagnosis during the first wave of COVID‐19. According to studies that used a more recent pandemic cohort (up to June 2021) those diagnosed later in the pandemic continued to demonstrate stable early survival.^23^, ^24^, ^25^ While these results seem optimistic, they also imply many cancer cases that are, in theory, at a more advanced stage, are still missing. Indeed, we estimated nearly 17,000 cancers were undetected in Ontario as of October 2021, and that during January–October 2021 the incidence of cancer actually decreased by 0.9% for each week.^42^ These ‘missing’ cancer cases will result in excess cancer mortality in this decade.^11^, ^12^, ^13^, ^14^ Policymakers must prioritize interventions to quickly ramp up cancer detection, including public health campaigns for cancer screening and hospital campaigns that invite patients to attend appointments sooner.

Our analysis was subject to the usual limitations of administrative data in being able to control for confounders, particularly the lack of cancer staging data, which typically require 2–3 years after diagnosis to mature within our cancer registry. Existing literature from the Unites States, Australia, and Europe suggested the pandemic‐associated upshift in cancer stage may be smaller than expected^20^, ^24^, ^25^, ^26^, ^28^; this should be confirmed with data from other jurisdictions. Next, we might have undercounted the death of cancer patients during the pandemic. Notably, patients who died of COVID‐19 or died outside of hospital and never had a postmortem examination leading to a cancer diagnosis would not be captured. Prior to the pandemic, these patients would have been coded as being dead on the date of cancer diagnosis; hence, we constructed a conservative study cohort by excluding all patients who died on the date of cancer diagnosis. Future studies with more robust vital statistics data should examine the potential bias arisen from this issue. Furthermore, although we followed the entire cohort for 1 year after diagnosis, for some cancers (such as breast cancer), this will not be long enough to detect a meaningful survival difference. Also, we did not conduct any focused investigation on rarer malignancies (such as leukemia). Finally, Ontario's single‐payer healthcare system means our findings may not be generalizable to other jurisdictions.

## 5 CONCLUSIONS

We used linked administrative data from a large province wide population‐based cohort to assess overall survival over the first year among adults diagnosed with cancer during the pandemic (March 15–December 31, 2020) and in pre‐pandemic (March 15–December 31, 2018/2019). Overall, no difference in 1‐year survival was found for patients diagnosed during the pandemic. Individually, a small but significantly higher survival was observed for hepatobiliary and lung cancers. Melanoma was the only cancer type associated with worse survival during the pandemic. These findings demonstrate the complex effect of the pandemic on the trajectory of cancer care that may not have necessarily translated into a decrease in early survival for those diagnosed early with COVID‐19. Efforts should be made to find the “missing” cancer patients and closely monitor the status of existing patients.

## AUTHOR CONTRIBUTIONS

**Rui Fu:** Conceptualization (equal); investigation (equal); methodology (equal); writing – original draft (lead); writing – review and editing (equal). **Rinku Sutradhar:** Conceptualization (equal); data curation (equal); investigation (equal); methodology (equal); supervision (equal); writing – review and editing (equal). **Qing Li:** Conceptualization (equal); data curation (equal); formal analysis (equal); methodology (equal); writing – review and editing (equal). **Pabiththa Kamalraj:** Conceptualization (equal); investigation (equal); project administration (equal); writing – review and editing (equal). **Anna Dare:** Conceptualization (equal); investigation (equal); writing – review and editing (equal). **Timothy Hanna:** Conceptualization (equal); investigation (equal); writing – review and editing (equal). **Kelvin Chan:** Conceptualization (equal); investigation (equal); writing – review and editing (equal). **Jonathan C Irish:** Conceptualization (equal); investigation (equal); writing – review and editing (equal). **Natalie Coburn:** Conceptualization (equal); investigation (equal); writing – review and editing (equal). **Juile Hallet:** Conceptualization (equal); investigation (equal); writing – review and editing (equal). **Simron Singh:** Conceptualization (equal); investigation (equal); writing – review and editing (equal). **Ambica Parmar:** Conceptualization (equal); investigation (equal); writing – review and editing (equal). **Craig C Earle:** Conceptualization (equal); investigation (equal); writing – review and editing (equal). **Lauren Lapointe‐Shaw:** Conceptualization (equal); investigation (equal); writing – review and editing (equal). **Monika Krzyzanowska:** Conceptualization (equal); investigation (equal); writing – review and editing (equal). **Alexander V. Louie:** Conceptualization (equal); investigation (equal); writing – review and editing (equal). **Alyson Mahar:** Conceptualization (equal); investigation (equal); methodology (equal); writing – review and editing (equal). **David Urbach:** Conceptualization (equal); investigation (equal); writing – review and editing (equal). **Daniel McIsaac:** Conceptualization (equal); investigation (equal); writing – review and editing (equal). **Danny J. Enepekides:** Conceptualization (equal); investigation (equal); writing – review and editing (equal). **David Gomez:** Conceptualization (equal); investigation (equal); writing – review and editing (equal). **Nicole J Look Hong:** Conceptualization (equal); investigation (equal); writing – review and editing (equal). **Jill Tinmouth:** Conceptualization (equal); investigation (equal); methodology (equal); writing – review and editing (equal). **Antoine Eskander:** Conceptualization (equal); funding acquisition (equal); investigation (equal); methodology (equal); supervision (equal); writing – review and editing (equal).

## FUNDING INFORMATION

This study was supported by ICES, which is funded by an annual grant from the Ontario Ministry of Health and the Ministry of Long‐Term Care. This study also received funding from a Sunnybrook Research Institute and Sunnybrook Foundation COVID‐19 Response Grant and a Canadian Institutes of Health Research Operating Grant #179892. The funders had no role in the design and conduct of the study, nor the decision to prepare and submit the manuscript for publication.

## CONFLICT OF INTEREST STATEMENT

Dr. Antoine Eskander reports research funding from Merck (2019) and consultant fee from Bristol‐Myers Squibb (2019). All other listed authors have disclosed that they have not received any financial consideration from any person or organization to support the preparation, analysis, results, or discussion of this article.

## Supporting information


Data S1:

## Data Availability

The dataset from this study is held securely in coded form at ICES. While legal data sharing agreements between ICES and data providers (e.g., healthcare organizations and government) prohibit ICES from making the dataset publicly available, access may be granted to those who meet pre‐specified criteria for confidential access, available at www.ices.on.ca/DAS (email: das@ices.on.ca). The full dataset creation plan and underlying analytic code are available from the authors upon request, understanding that the computer programs may rely upon coding templates or macros that are unique to ICES and are therefore either inaccessible or may require modification.
