# Supplementary material for: Early survival for patients newly diagnosed with cancer during COVID‐19 in Ontario, Canada: A population‐based cohort study
Source: Cancer Med. 2023 Mar 31;12(10):11849–59. doi: 10.1002/cam4.5861 (PMC10242323; doi:10.1002/cam4.5861)
Supplement: Supplementary file 1 — Data S1: [file CAM4-12-11849-s001.docx]

**Early survival for patients newly diagnosed with cancer during COVID-19 in Ontario, Canada: a population-based cohort study**

**Supplementary Appendix**

**Appendix I** Data sources

| **Database** | **Description** |
| --- | --- |
| **Ontario Cancer Registry (OCR)** | The OCR is the provincial registry of all incident cancer diagnoses in Ontario with a capture rate of 96%. Information included in the registry: cancer topography and morphology/histology, and details on diagnosis (e.g., types of contributing information to the diagnosis, dates), and patients who have died of cancer. |
| **Ontario Health Insurance Plan (OHIP) Claims Database** | The OHIP claims database contains all physician billing records including information on diagnoses and services provided, such as receipt of chemotherapy and radiotherapy.  Chemotherapy: G339, G345, G359, G381, G281, G382, G388  Radiotherapy: X310, X311, X312, X313, X302, X304, X305, X306, X322, X323, X334, X324, X325, X326, X327, X335, X328, X329, X332, X336, X330 |
| **Discharge Abstract Database (DAD) and Same Day Surgery (SDS)** | The DAD and SDS are both data holdings of the Canadian Institute for Health Information (CIHI) that capture administrative, clinical, and demographic information on hospital discharges (including deaths, sign-outs, and transfers) and day surgery. |
| **Registered Persons Database (RPDB)** | The RPDB is an ICES database derived from all administrative data sources and provides demographic data including age, patient residence, vital status, date of last contact with the healthcare system, and OHIP eligibility. |
| **Immigration, Refugees and Citizenship Canada (IRCC) Permanent Residents database** | The IRCC Permanent Resident Database (with data from January 1985 to May 2017) includes records of individuals who immigrated to Ontario during this period. |
| **Ontario Marginalization Index (ON-MARG)** | The ON-MARG is a specialized database using census data to profile relative area-level marginalization dependency, deprivation, ethnic concentration, and instability at various geographic levels in Ontario. The material deprivation dimension captures the proportion of population that is without a high school degree, unemployed, low-income, single-parent families, receiving government transfer payments, and living in dwelling in need of a major repair. |
| **Ontario Registrar General (ORG)** | The ORG contains gold standard vital status data for all Ontarians. According to the Vital Statistics Act, it is mandatory to register all deaths occurring in the province. |

**Appendix II** Identifying cancer diagnosis records from the Ontario Cancer Registry (OCR)

| **Cancer site** | **ICD-O-3 code** |
| --- | --- |
| **Breast** | C50 |
| **Central nervous system** | C70.0, C70.1, C70.9, C71, C72 |
| **Colorectal** | C17, C18, C19.9, C20.9, C21.0, C21.1, C21.2, C21.8 |
| **Cervical** | C53.0, C53.1, C53.8, C53.9 |
| **Endocrine** | C73.9, C74.0, C74.1, C74.9, C75 |
| **Esophagus** | C15 |
| **Genitourinary** | C60, C62, C64, C65, C66, C67, C68 |
| **Gynecological exclude cervical** | C51, C52, C54, C55, C56, C57 |
| **Head and neck** | C10.0, C10.1, C10.2, C10.3, C10.4, C10.8, C10.9, C01.9, C02.0, C02.1, C02.2, C02.3, C02.4, C02.8, C02.9, C03.0, C03.1, C03.9, C04.0, C04.1, C04.8, C04.9, C05.0, C05.1, C05.2, C05.8, C05.9, C06.0, C06.1, C06.2, C06.8, C06.9, C07.9, C08.0, C08.1, C08.8, C08.9, C09.0, C09.1, C09.8, C09.9, C11.0, C11.1, C11.2, C11.3, C11.8, C11.9, C12.9 , C14.0, C14.2, C14.8, C76.0, C06.9, C14.8, C32.0, C32.1, C32.3, C32.8, C32.9, C13.0, C13.1, C13.2, C13.8, C13.9, C00.0, C00.1, C00.2, C00.3, C00.4, C00.5, C00.6, C00.8, C00.9, C14.8, C44.0 |
| **Hepatobiliary** | C22.0, C22.1, C23, C24, C25 |
| **Lung** | C34 |
| **Lymphoma** | All sites with morphology codes 9650-9667; 9590-9597, 9670-9671, 9673, 9675, 9678-9680, 9684, 9687, 9689-9691, 9695, 9698-9702, 9705, 9708-9709, 9714-9719, 9727-9729; all sites other than C42.0, C42.1, C42.4 with morphology codes 9823, 9827 |
| **Prostate** | C61.9 |
| **Sarcoma** | C00.0, C00.1, C00.3, C00.5, C00.9, C01.9 to C02.3, C02.8 to C03.1, C03.9, C04.0, C04.9, C.05.0, C05.1, C05.9, C06.0, C06.2, C06.9, C07.9, C08.0, C08.9, C09.0, C09.9, C10.3, C10.9, C11.0 to C11.3, C11.8, C11.9, C13.0, C13.1, C13.8, C13.9, C14.0, C14.8, C15.0, C15.3, C15.4, C15.5, C15.9, C16.0 to C16.6, C16.8 to C17.3, C17.8 to C18.9, C19.9, C20.9, C22.0, C22.1, C23.9 to C24.1, C24.9 to C25.2, C25.9, C30.0, C30.1, C31.1 to C31.3, C31.8 to C32.3, C32.9, C33.9 to C34.3, C34.8, C34.9, C37.9 to C38.3, C40.1 to C40.3, C40.8 to C41.4, C41.9, C42.1 to C42.4, C44.0 to C44.9, C47.0 to C47.9, C49.0 to C49.9, C50.0 to C512, C51.8, C51.9, C52.9 to C53.1, C53.8 to C54.3, C54.8, C54.9, C56.9 to C57.4, C57.7 to C57.9, C60.0 to C60.2, C60.9, C61.9 to C62.1, C62.9 to C63.2, C63.7 to C63.9, C649., C65.9, C66.9 to C68.0, C68.8, C69.0, C69.3, C69.6, C69.8, C70.0, C70.1, C70.9 to C72.0, C72.5, C72.9, C73.9 to C74.1, C74.9, C75.5, C77.0 to C77.9 with morphology codes 803*, 831*, 871*, 880*-885*, 890*-900*, 912*, 914*, 917*-919*, 922*-924*, 926*, 933*, 944*, 948*, 953*, 958*, 974*-975*, 993* |
| **Melanoma** | C44 with morphology codes 8720-8790 |
| **Skin** | C44.2-C44.9 |
| **Stomach** | C16 |
| **Ophthalmologic** | C69 |
| **Paraneoplastic neurological syndromes** | C47 |
| **Other** | C26.0, C26.8, C26.9, C30, C31, C32.2, C33.9, C37.9, C38, C39, C40, C41, C42.0-C42.4, C44.1, C48, C49, C58.9, C63, C76, C80.9 |

To select invasive cancers, we included diagnoses with an ICD-O-3 morphology code with a fifth digit 3/6/9 (3-malignant, primary site; 6-malignant, metastatic site, secondary site; 9-malignant, uncertain whether primary or metastatic site). Other diagnoses were deemed to be in-situ and thus were excluded from this analysis.

**Figure S1** A flow diagram documenting the creation of the study cohort (n =179,746)

All new cancer diagnoses that occurred in Ontario, Canada during the 3 yearly periods (March 15—December 31 in 2018, 2019, and 2020)*

N=193,427

Patients were excluded for:

- Incorrectly coded death date (n=99)
- Age < 18 at the time of diagnosis (n=1,166)
- Incorrectly coded sex or being a non-Ontario resident (n=98)
- Two or more invasive cancer types diagnosed on the same day (n=814)
- Died on the date of cancer diagnosis (n=1,624)

Only select the first diagnosis for each patient in these periods

N=183,547

Final study cohort

N=179,746

* Patients need to have at least one invasive cancer (morphology fifth digit 3/6/9) diagnosed during these yearly periods to enter the cohort.

**Appendix III** Association between the pandemic and 1-year overall survival in newly diagnosed cancer patients for each cancer type (n=179,746)

| **Cancer type** | **HR of 1-year all-cause mortality for patients in the pandemic cohort vs pre-pandemic cohort** | **95% CI** |
| --- | --- | --- |
| Breast | 1·09 | 0·95–1·24 |
| Colorectal | 1·00 | 0·93–1·07 |
| Endocrine | 1·11 | 0·80–1·55 |
| Esophagus | 0·95 | 0·83–1·09 |
| Genitourinary | 1·00 | 0·91–1·10 |
| Gynecologic excluding cervical | 0·96 | 0·85–1·09 |
| Head and neck | 1·04 | 0·91–1·18 |
| Hepatobiliary | 0·85 | 0·80–0·90 |
| Lung | 0·95 | 0·91–0·99 |
| Lymphoma | 1·04 | 0·94–1·15 |
| Melanoma | 1·25 | 1·05–1·49 |
| Prostate | 1·03 | 0·89–1·19 |
| Sarcoma | 1·09 | 0·91–1·32 |
| Skin | 0·87 | 0·54–1·39 |
| Stomach | 1·01 | 0·91–1·13 |
| Other | 1·00 | 0·95–1·06 |

We report the hazard ratios and associated 95% confidence intervals of 1-year all-cause mortality from a multivariable Cox proportional hazards model (Model 3), where interaction of the pandemic indicator (pandemic vs. pre-pandemic) with each cancer type was included. Model 3 also comprised the following patient-level covariates: age at cancer diagnosis, sex, rurality, immigration status, material deprivation, comorbidity measured by the Elixhauser Comorbidity Index, and first cancer treatment received within the first post-diagnosis year modelled as a time-varying covariate. “Other” includes cancers of the central nervous system, cervix, ophthalmologic, paraneoplastic neurological syndromes, and those with ill-defined or unknown primary sites.

**Abbreviations:** HR, hazard ratio; CI, confidence interval.

**Appendix IV Sensitivity analysis: excluding patients who died on the date of cancer diagnosis**

**Figure S2** Survival in new cancer patients within the first year after diagnosis (n = 181,370)


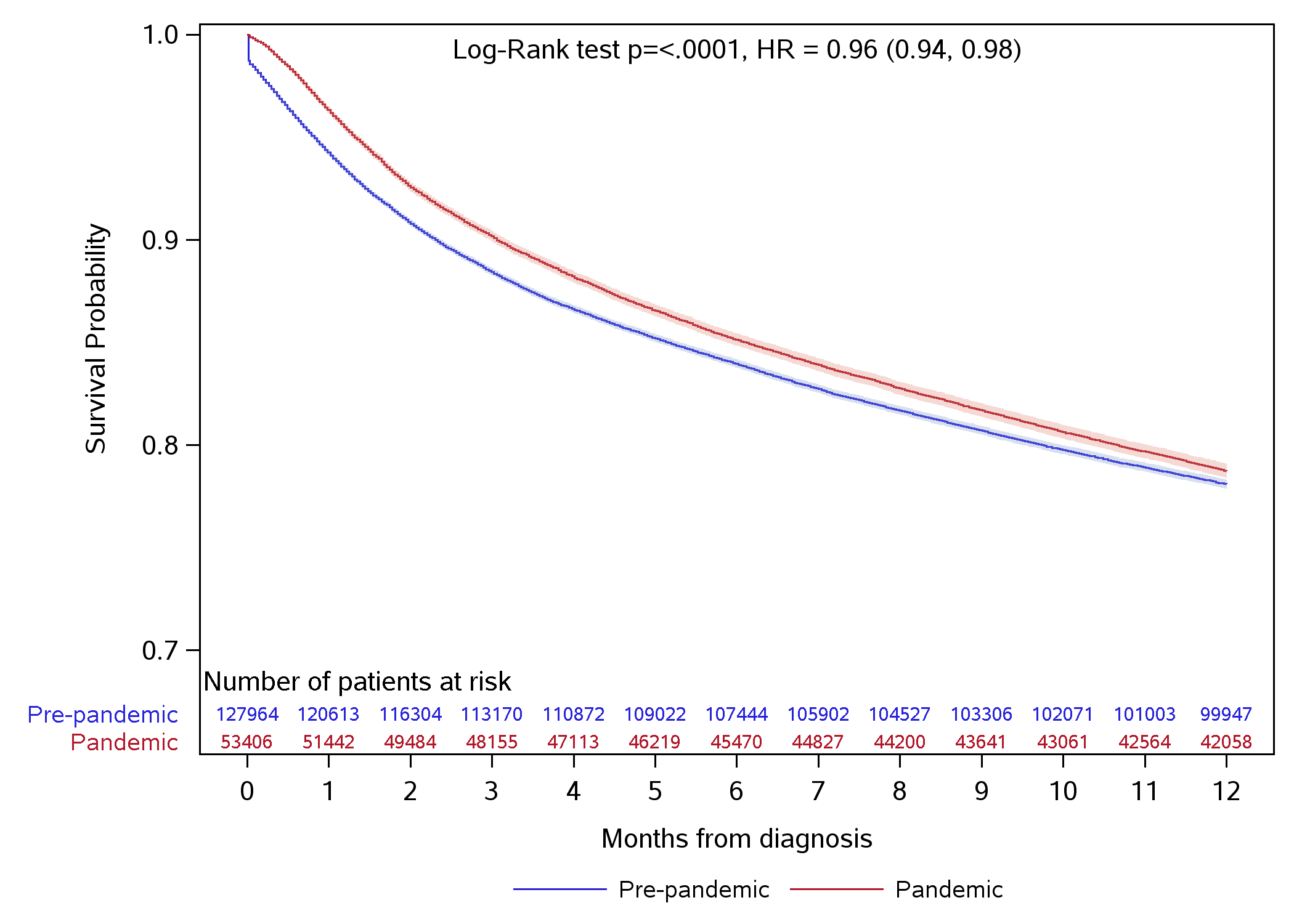


We defined the pandemic cohort to comprise patients diagnosed with cancer from March 15–December 31, 2020, the first 9.5 months of pandemic control in Ontario, Canada, since hospitals had been advised to discontinue nonemergent and elective procedures. Corresponding dates in 2018 and 2019 were used to construct a pre-pandemic cohort. This figure shows the Kaplan-Meier estimates of 1-year overall survival (with 95% confidence interval) to be significantly higher in patients who were diagnosed in the pandemic compared to the pre-pandemic.

**Table S1** Characteristics of patients at time of diagnosis (n=181,370)

| **Variables** | **Pre-pandemic cohort (N=127,964)** | **Pandemic cohort (N=53,406)** | **Standardized difference** |
| --- | --- | --- | --- |
| **Age at diagnosis,** year | 66·89 ± 14·17 | 66·43 ± 14·16 | 0·03 |
| **Female** | 64,924 (50·7%) | 27,215 (51·0%) | 0 |
| **Rural^1^** | 16,489 (12·9%) | 7,061 (13·2%) | 0·01 |
| **Immigrant** | 16,091 (12·6%) | 7,014 (13·1%) | 0·02 |
| **Region** |  |  |  |
| Central | 36,925 (28·9%) | 15,393 (28·8%) | 0 |
| East | 33,102 (25·9%) | 13,878 (26·0%) | 0 |
| North | 8,978 (7·0%) | 3,887 (7·3%) | 0·01 |
| Toronto | 10,094 (7·9%) | 4,055 (7·6%) | 0·01 |
| West | 38,865 (30·4%) | 16,193 (30·3%) | 0 |
| **Material deprivation quintile^1,2^** |  |  |  |
| 1 (least deprived) | 27,904 (21·8%) | 11,747 (22·0%) | 0 |
| 2 | 26,063 (20·4%) | 11,033 (20·7%) | 0·01 |
| 3 | 24,817 (19·4%) | 10,323 (19·3%) | 0 |
| 4 | 24,256 (19·0%) | 10,102 (18·9%) | 0 |
| 5 (most deprived) | 23,841 (18·6%) | 9,726 (18·2%) | 0·01 |
| **Comorbidity grouping^3^** |  |  |  |
| 0 | 12,231 (9·6%) | 5,314 (10·0%) | 0·01 |
| 1 | 10,429 (8·1%) | 4,430 (8·3%) | 0·01 |
| 2 | 7,827 (6·1%) | 3,098 (5·8%) | 0·01 |
| 3+ | 11,377 (8·9%) | 4,320 (8·1%) | 0·03 |
| No hospitalization | 86,100 (67·3%) | 36,244 (67·9%) | 0·01 |
| **Cancer type** |  |  |  |
| Breast | 18,278 (14·3%) | 7,654 (14·3%) | 0 |
| Central nervous system | 1,607 (1·3%) | 759 (1·4%) | 0·01 |
| Cervical | 972 (0·8%) | 423 (0·8%) | 0 |
| Colorectal | 14,036 (11·0%) | 5,869 (11·0%) | 0 |
| Endocrine | 4,624 (3·6%) | 1,780 (3·3%) | 0·02 |
| Esophagus | 1,345 (1·1%) | 588 (1·1%) | 0 |
| Genitourinary | 9,063 (7·1%) | 3,862 (7·2%) | 0·01 |
| Gynecologic excluding cervical | 6,949 (5·4%) | 3,215 (6·0%) | 0·03 |
| Head and neck | 4,027 (3·1%) | 1,808 (3·4%) | 0·01 |
| Hepatobiliary | 6,503 (5·1%) | 2,824 (5·3%) | 0·01 |
| Lung | 15,862 (12·4%) | 6,445 (12·1%) | 0·01 |
| Lymphoma | 6,472 (5·1%) | 3,019 (5·7%) | 0·03 |
| Melanoma | 6,538 (5·1%) | 2,353 (4·4%) | 0·03 |
| Ophthalmologic | 55 (0·0%) | 16 (0·0%) | 0·01 |
| PNS | 21 (0·0%) | 10 (0·0%) | 0 |
| Prostate | 14,194 (11·1%) | 5,270 (9·9%) | 0·04 |
| Sarcoma | 1,979 (1·5%) | 883 (1·7%) | 0·01 |
| Skin | 442 (0·3%) | 137 (0·3%) | 0·02 |
| Stomach | 2,202 (1·7%) | 938 (1·8%) | 0 |
| Other | 12,795 (10·0%) | 5,553 (10·4%) | 0·01 |

Data are n (%) or mean ± standard deviation (for age). We defined the pandemic cohort to include patients diagnosed with cancer during March 15–December 31, 2020 to coincide with the first 9.5 months since hospitals had been advised to discontinue nonemergent and elective procedures. Corresponding dates in 2018 and 2019 were used to construct a pre-pandemic cohort. A standardized difference of 0·1 or greater indicated a significant imbalance in the distributions.

^1^ Missing data of these variables were less than 1·0% of the study cohort and the distributions of missingness did not differ by COVID-19 period (both standardized differences < 0·02).

^2^ Material deprivation is a composite measure of socioeconomic status that includes the proportion of the population that is without a high school degree, single-parent families, unemployed, low-income, receiving government transfer payments, and living in a dwelling in need of a major repair.

^3^ The Elixhauser Comorbidity Index was computed based on health administrative data 5 years leading to the date of cancer diagnosis.

**Abbreviations:** PNS, Paraneoplastic neurological syndromes.

**Table S2** First cancer treatment received within the first year after diagnosis (n=181,370)

| **Variables** | **Pre-pandemic (N=127,964)** | **Pandemic (N=53,406)** | **Standardized difference** |
| --- | --- | --- | --- |
| **First cancer treatment** |  |  |  |
| Untreated | 33,867 (26·5%) | 11,669 (21·8%) | 0·11 |
| Systemic therapy | 21,827 (17·1%) | 10,939 (20·5%) | 0·09 |
| Radiotherapy | 19,139 (15·0%) | 8,828 (16·5%) | 0·04 |
| Surgery | 53,131 (41·5%) | 21,970 (41·1%) | 0·01 |
| **Time to first treatment, day** |  |  |  |
| Mean ± SD | 51·54 ± 57·49 | 45·63 ± 52·72 | 0·11 |
| Median (IQR) | 37·0 (15·0–66·0) | 33·0 (13·0–58·0) | 0·11 |

Time to first treatment was only calculated for the sub-cohort of patients that was treated by any means within the first year after cancer diagnosis. The pandemic cohort included patients newly diagnosed with cancer from March 15 to December 31, 2020 and the pre-pandemic cohort included patients diagnosed during the corresponding dates in 2018 and 2019.

**Abbreviations:** SD, standard deviation; IQR, interquartile range.

**Table S3** Results of the Cox proportional hazards regression analysis showing the association between COVID-19 pandemic period and 1-year survival in new cancer patients (n=181,370)

| **Variables** | **HR** | **95% CI** | **p-value** |
| --- | --- | --- | --- |
| **Univariable model** |  |  |  |
| Pandemic vs pre-pandemic | 0·96 | 0·94–0·98 | <0·01 |
| **Multivariable model (Model 1)** |  |  |  |
| Pandemic vs pre-pandemic | 0·93 | 0·91–0·96 | <0·01 |
| **Multivariable model (Model 2)** |  |  |  |
| Pandemic vs pre-pandemic | 0·92 | 0·90–0·94 | <0·01 |
| Status of first cancer treatment, vs untreated  Systemic therapy  Radiotherapy  Surgery | 1·37  1·68  0·42 | 1·33–1·41  1·62–1·73  0·40–0·44 | <0·01  <0·01  <0·01 |
| Age at cancer diagnosis, each 10-year increase | 1·52 | 1·51–1·54 | <0·01 |
| Female vs male | 0·94 | 0·92–0·96 | <0·01 |
| Rural vs urban | 1·08 | 1·05–1·11 | <0·01 |
| Immigrants vs non-immigrants | 0·84 | 0·81–0·87 | <0·01 |
| Material deprivation, vs 5 – most deprived  1 – least deprived  2  3  4 | 0·77  0·83  0·87  0·91 | 0·75–0·80  0·80–0·85  0·84–0·90  0·88–0·94 | <0·01  <0·01  <0·01  <0·01 |
| Comorbidity, vs no hospitalization  0  1  2  3+ | 1·02  1·17  1·29  1·55 | 0·99–1·06  1·13–1·21  1·25–1·34  1·50–1·59 | 0·24  <0·01  <0·01  <0·01 |
| Cancer type, vs breast cancer  Colorectal  Endocrine  Esophagus  Genitourinary  Gynecologic excluding cervical  Head and neck  Hepatobiliary  Lung  Lymphoma  Melanoma  Prostate  Sarcoma  Skin  Stomach  Other^1^ | 3·69  1·29  7·51  3·83  3·10  2·96  11·46  6·85  2·84  1·09  0·67  2·56  1·49  7·18  4·32 | 3·45–3·95  1·10–1·52  6·88–8·19  3·55–4·13  2·86–3·37  2·71–3·22  10·72–12·25  6·42–7·31  2·63–3·06  0·98–1·20  0·61–0·73  2·30–2·86  1·21–1·84  6·63–7·78  4·04–4·61 | <0·01  0·02  <0·01  <0·01  <0·01  <0·01  <0·01  <0·01  <0·01  0·11  <0·01  <0·01  <0·01  <0·01  <0·01 |

Model 1 adjusts for all covariates in Model 2 except for first cancer treatment modality as a time-varying covariate. ^1^Other includes cancers of the central nervous system, cervix, ophthalmologic, paraneoplastic neurological syndromes, and those with ill-defined or unknown primary sites.

**Abbreviations:** HR, hazard ratio; CI, confidence interval.

**Table S4** COVID-19 pandemic association with 1-year overall survival for each cancer type (n=181,370)

| **Cancer type** | **HR of 1-year all-cause mortality for patients in the pandemic cohort vs pre-pandemic cohort** | **95% CI** |
| --- | --- | --- |
| Breast | 0·97 | 0·85–1·10 |
| Colorectal | 0·93 | 0·870–0·999 |
| Endocrine | 1·01 | 0·73–1·40 |
| Esophagus | 0·94 | 0·82–1·07 |
| Genitourinary | 0·94 | 0·85–1·03 |
| Gynecologic excluding cervical | 0·91 | 0·80–1·02 |
| Head and neck | 0·97 | 0·86–1·10 |
| Hepatobiliary | 0·82 | 0·77–0·87 |
| Lung | 0·91 | 0·87–0·95 |
| Lymphoma | 1·02 | 0·92–1·12 |
| Melanoma | 1·23 | 1·03–1·46 |
| Prostate | 0·92 | 0·80–1·06 |
| Sarcoma | 1·09 | 0·90–1·31 |
| Skin | 0·86 | 0·54–1·39 |
| Stomach | 0·99 | 0·89–1·11 |
| Other | 0·93 | 0·88–0·98 |

We report the hazard ratios and associated 95% confidence intervals of 1-year all-cause mortality from a multivariable Cox proportional hazards model (Model 3), where interaction of the pandemic indicator (pandemic vs. pre-pandemic) with each cancer type was included. Model 3 also comprised the following patient-level covariates: age at cancer diagnosis, sex, rurality, immigration status, material deprivation, comorbidity measured by the Elixhauser Comorbidity Index, and first cancer treatment received within the first post-diagnosis year modelled as a time-varying covariate. “Other” includes cancers of the central nervous system, cervix, eyes, paraneoplastic neurological syndromes, and those with ill-defined or unknown primary sites.

**Abbreviations:** HR, hazard ratio; CI, confidence interval.

**Appendix V Systematic review of existing literature**

We searched MEDLINE and EMBASE for peer-reviewed publications from inception (1946) to February 9, 2023 that compared the survival outcomes of adults newly diagnosed with cancer before and after the start of the COVID-19 pandemic. Snowballing search was also conducted to identify more studies.

**Ovid MEDLINE: Epub Ahead of Print, In-Process & Other Non-Indexed Citations, Ovid MEDLINE® Daily and Ovid MEDLINE® <1946-Current>**

1 exp Neoplasms/ 3791178

2 ("cancer" or "malignancy" or oncolog*).ti,ab. 2236049

3 ("new" or "incident" or diagnos*).ti,ab. 5687104

4 exp Survival Analysis/ or exp Survival/ 336627

5 (surviv* or survival or death or mortality or die*).ti,ab. 3555692

6 (exp coronavirus/ or coronavirus*.mp.) and (wuhan or Beijing or shanghai or 2019-nCOV or nCoV or COVID-19 or SARS-CoV-2).mp. 206429

7 Coronavirus*.ti. or (novel coronavirus*.mp. and (exp china/ or china.mp.)) or ((pneumonia.mp. or exp pneumonia/) and Wuhan.mp.) 35723

8 ("COVID-19" or "2019-nCoV" or "SARS-CoV-2").mp. or exp Coronavirus Infections/ 341554

9 (1 or 2) and 3 1138321

10 4 or 5 3627311

11 6 or 7 or 8 346791

12 9 and 10 and 11 1522

13 limit 12 to (english language and humans and yr="1946 - Current") 1037

**Embase Classic+Embase <1947 to Current>**

1 exp malignant neoplasm/ 4069717

2 ("cancer" or "malignancy" or oncolog*).ti,ab. 3306281

3 ((new or incident or diagnos*) adj5 (cancer or malignancy)).ti,ab. 310158

4 exp survival analysis/ or cancer survival/ or exp overall survival/ or exp survival/ 1428573

5 (surviv* or survival or death or mortality or die*).ti,ab. 5173658

6 exp coronavirus disease 2019/ 314830

7 ("covid 19" or (covid adj2 "19") or (sars adj2 "cov 2") or "2019 ncov" or ((wuhan or hubei) and coronavirus*)).ti,ab. 356275

8 (1 or 2) and 3 310158

9 4 or 5 5413176

10 6 or 7 392609

11 (8 and 9 and 10) 1121

12 limit 11 to (human and english language and yr="1946 - Current") 1089

| **Source** | **Country** | **Cancer types examined** | **Cohort description** | **Survival outcomes** | **Was the analysis adjusted?** | **Pandemic effect on survival?** | **Main findings** |
| --- | --- | --- | --- | --- | --- | --- | --- |
| Khan 2023 | USA | Esophageal | 307 adults newly diagnosed at a single centre in Mar-Dec 2020 vs. 2019 | 1-year OS | Unadj | NONE | 1y mortality: 76.4% v 83.7% (p=0.58)  No TMN stage shift (p=0.34) |
| Aparicio 2023 | France | Digestive system | 7881 older adults>65 newly treated in 30 hospitals in 2020 vs. 2018-2019 | 10-month OS (fully observed) | Adj | NONE  (Unless hospitalized for COVID) | No difference for pre-lockdown, lockdown and post-lockdown periods in 2020 vs. 2018/2019 |
| Abu-Freha  2022 | Israel | CRC and gastric | 438 adults newly diagnosed in 2019 and 2020 at a university hospital | All-cause death (11m-23m from date of diagnosis) | Unadj | NONE | All-cause death:  CRC (n=378): 31% v 24% (p=.2)  No stage shift (all p>.05)  No diff in time-to-surgery (p=.19)  Gastric (n=60): 50% v 50% (p>.9)  No stage shift (all p>.05) |
| Geh 2022 | UK | Liver (hepatocellular carcinoma) | 310 adults newly diagnosed and referred in North East England/Cumbria from Mar 2019-Feb 2020 and same period in 2020-2021 | OS (max follow-up=35m) | Unadj | NONE (Unless w ICC) | Log-rank p=.987  ICC (n=140) survival log-rank p=.028 (pandemic is worse) |
| Chen 2022 | Australia | CRC | 1,609 adults newly diagnosed in six CRC units in Melbourne from July 1, 2018-June 30, 2019 and same period in 2020-2021 | Inpatient death (unknown follow-up duration) | Unadj | BETTER | Inpatient death: 2.7% v 0.8% (p=.019)  30d readmission: 10% v 3.5% (p<.001)  No T-stage shift (p=.4), both N-/M-stage migrated upwards (both p<.03) |
| Keogh 2022 | Canada | NSCLC | 695 adults newly diagnosed and surgically treated at a single centre from Jan 2019-Feb 2020 and from Mar 2020-Feb 2021 | OS (unknown follow-up duration) | Unadj | NONE | Mortality: 4.8% v 6.2% (p=.4)  No TNM stage migration (all p>.3) |
| Eklöv 2022 | Sweden | Colon | 7,980 adults newly diagnosed in Sweden from April-May in 2019 and 2020 and treated by surgery | 30d post-op death | Unadj | NONE | 30d mortality: 1.4% v 1.1% (p=.36)  Shorter mean time-to-surgery:  33d v 29d (p<.01)  Increased neoadjuvant chemo: 3.5% v 5.1% (p<.01) |
| Kempf 2022 | France | CRC | 4,685 adults newly referred to 1 of 27 teaching hospitals from Jan 1-Dec 31 in 2018-2019 and 2020 | 1-year OS after diagnosis | Unadj | NONE  (Unless infected w COVID) | Surgical resection:  94% v 93% v 76% (w COVID)  Other anti-cancer treatments:  64% v 66% v 27% (w COVID) |
| Tejedor-Tejada 2022 | Spain | Pancreatic | 25 adults newly referred to a single center from Mar-Sep 2019 and 2020 | 1-year OS after referral | Unadj | NONE | Unadj 1-year death:  50% v 61.5% (p=0.449)  Stage III/IV: 30.8% v 53.8% (p<.01) |
| Aparicio  2022 | France | Digestive system (CRC, oesophagus, gastric, pancreatic, small intestine, anus, bile duct, hepatocellular) | 7,882 older adults>65 newly treated in 1 of 30 hospitals in Paris from Jan 1-Aug 30 in 2018, 2019 and 2020. | 3m OS after hospital admission | Unadj | NONE | Survival comparing 2018/19 v 2020:  Lockdown period (Mar 16-May 10):  78.9%/81.1% v 81.7%, p=.57  Post-lockdown (May 11-Aug 30):  82.0%/79.2% v 82.8%, p=.06 |
| Morais  2021 | Portugal | Esophagus, gastric, CRC, pancreas, lung, skin-melanoma, breast, cervix, prostate | 2,072 adults newly diagnosed at a single centre from Mar 2-Jul 1 in 2019 and 2020, followed up to Oct 31. | ‘Short-term’ OS after diagnosis | Adj | NONE | Unadj HR=1.51 (1.20-1.91)  Age/stage adj HR=1.10 (0.91-1.46)  Propensity score HR=1.10 (0.86-1.40) |
| Guven  2021 | Turkey | Breast, CRC, lung, cervix, melanoma, pancreatic, HNC, gastric, sarcoma, GU, CNS, ovary, endometrial, other | 2,208 adults newly referred to a single outpatient Med Onc clinic from Mar 1-Dec 31 in 2019 and 2020. | 90d OS after first referral | Unadj | WORSE | Presenting with advanced stage or metastatic: 39% v 50%  (Unadj OR=1.55, p<.01)  90d death: 6.6% v 10.5%  (Unadj OR=1.66, p<.01) |
| Paluri  2022 | USA | Metastatic pancreatic ductal adenocarcinoma (mPDAC) | 1,719 adults newly diagnosed and recorded in the Flatiron Health Database (~280 cancer clinics) from Mar 1-Sep 30 in 2019 and 2020. | 180d OS after diagnosis | Unadj | WORSE | Unadj Kaplan-Meier survival (p<.01)  60d: 86.2% v 82.8%  90d: 77.0% v 71.4%  120d: 71.0% v 62.1%  180d: 61.4% v 51.4% |
| Rottoli  2022 | Italy | CRC | 3,236 adults operated for CRC in 20 treatment centres nationwide from Mar 1-Dec 31 in 2019 and 2020. | 30d death post-op | Unadj | NONE | Presenting with advanced stage (48.3% v 49.1%): aOR=1.01, p=.92  Receiving palliative surgery  (5.0% v 7.5%): aOR=1.46, p=.09  30d death: 1.7% v 2.4%, p=.15 |
| Smith  2021 | Denmark | CRC | 2,794 adults newly diagnosed in Denmark (2,236 stage I-III operated) from Mar 1-Aug 1 in 2019 and 2020 | 30d, 90d death post-op | Adj | NONE | 30d death: aHR=1.55, p=.12  90d death: aHR=1.07, P=.17 |

**Abbreviations:** ICC, intrahepatic cholangiocarcinoma; CRC, colorectal; OS, overall survival; adj, adjusted; btw, between; avg, average; HR, hazard ratio; HNC, head and neck; GU, genitourinary; CNS, central nervous system; NSCLC, non-small-cell lung cancer.
